# Generating Brain MRI with StyleGAN2-ADA: The Effect of the Training Set Size on the Quality of Synthetic Images

**DOI:** 10.1007/s10278-025-01536-0

**Published:** 2025-09-23

**Authors:** Matteo Lai, Mario Mascalchi, Carlo Tessa, Stefano Diciotti

**Affiliations:** 1 Department of Electrical, Electronic, and Information Engineering “Guglielmo Marconi” - DEI, University of Bologna, 47522 Cesena, Italy; 2 Experimental and Clinical Biomedical Sciences “Mario Serio”, University of Florence, 50139 Florence, Italy; 3 Unit of Radiology, Azienda USL Toscana Nord Ovest, Apuane Hospital, 54100 Massa, Italy; 4 Alma Mater Research Institute for Human-Centered Artificial Intelligence, University of Bologna, 40121 Bologna, Italy

**Keywords:** Brain MRI, Generative adversarial networks, Medical imaging, Synthetic data

## Abstract

The potential of deep learning for medical imaging is often constrained by limited data availability. Generative models can unlock this potential by generating synthetic data that reproduces the statistical properties of real data while being more accessible for sharing. In this study, we investigated the influence of training set size on the performance of a state-of-the-art generative adversarial network, the StyleGAN2-ADA, trained on a cohort of 3,227 subjects from the OpenBHB dataset to generate 2D slices of brain MR images from healthy subjects. The quality of the synthetic images was assessed through qualitative evaluations and state-of-the-art quantitative metrics, which are provided in a publicly accessible repository. Our results demonstrate that StyleGAN2-ADA generates realistic and high-quality images, deceiving even expert radiologists while preserving privacy, as it did not memorize training images. Notably, increasing the training set size led to slight improvements in fidelity metrics. However, training set size had no noticeable impact on diversity metrics, highlighting the persistent limitation of mode collapse. Furthermore, we observed that diversity metrics, such as coverage and β-recall, are highly sensitive to the number of synthetic images used in their computation, leading to inflated values when synthetic data significantly outnumber real ones. These findings underscore the need to carefully interpret diversity metrics and the importance of employing complementary evaluation strategies for robust assessment. Overall, while StyleGAN2-ADA shows promise as a tool for generating privacy-preserving synthetic medical images, overcoming diversity limitations will require exploring alternative generative architectures or incorporating additional regularization techniques.

**Supplementary Information:**

The online version contains supplementary material available at 10.1007/s10278-025-01536-0.

## Background

Deep learning (DL) techniques could benefit medical imaging by advancing personalized diagnosis and biomarker discovery. However, its potential is constrained by limited data availability, primarily due to privacy concerns [1], as well as the significant costs and time required to acquire medical images. A common strategy to overcome data scarcity is data augmentation, but augmented data often remains highly correlated with the original dataset [2]. An alternative approach is the generation of synthetic data using generative models [3–7]. These models aim to learn and replicate the statistical properties of the observed real data, creating a synthetic distribution [8]. High-quality synthetic data can allow analyses comparable to those conducted on real data while mitigating privacy failure risks. This facilitates broader data sharing, fosters open science, and accelerates research by providing a faster alternative to the lengthy process of collecting and curating real data.

Among the generative models, two techniques have emerged for medical image generation: generative adversarial networks (GANs) [9], and diffusion models [10]. Both generate high-quality images, but diffusion models tend to overfit training data [11, 12], while GANs can suffer from mode collapse, failing to capture the full diversity of the real data distribution [13, 14]. As these models rely on training DL architecture, their performance remains highly dependent on the size of the training dataset.

In this study, we focused on StyleGAN2-ADA, a state-of-the-art GAN introduced by Karras et al. [15], optimized for training with limited datasets, thanks to its adaptive discriminator augmentation (ADA). ADA dynamically adjusts the strength of the augmentations based on a heuristic that estimates the level of overfitting, applying a set of non-leaking augmentations that do not transfer to the generated images. To mitigate mode collapse, StyleGAN2-ADA implements two key mechanisms: minibatch standard deviation normalization in the discriminator and pixelwise feature vector normalization in the generator. Minibatch standard deviation normalization encourages the model to capture the full variation in the training data by ensuring that the statistics of generated and real images are aligned across minibatches. Meanwhile, pixelwise feature vector normalization prevents the magnitudes in the generator and discriminator from escalating due to adversarial competition, one of the primary triggers of mode collapse [16, 17].

StyleGAN2-ADA is recognized as one of the best-performing GAN architectures [18] and has demonstrated significant promise in medical imaging applications [19–22], particularly due to its optimization for limited-data scenarios. Its ability to generate high-quality synthetic images with small datasets has ensured its continued relevance in recent years [23–26]. However, its performance and reliability are influenced by two key factors: the size of the training dataset and the rigor of the evaluation metrics used to assess synthetic data quality. The authors of StyleGAN2-ADA explored the influence of training set size on image quality, though they limited their analysis to Fréchet Inception Distance (FID). Their results showed that while ADA improved performance compared to the StyleGAN2 baseline for small datasets, image quality remains dependent on training set size, particularly for smaller datasets. A recent work by Zoghby et al. evaluated the impact of training set size on brain MR image-to-image translation using a pix2pix GAN, finding that smaller datasets can yield results comparable to larger ones [27]. However, their dataset exhibited limited subject diversity due to the involvement of only 1,251 subjects, and employed a limited set of evaluation metrics, leaving open questions about how training set size affects broader aspects of synthetic data quality.

Evaluation metrics are pivotal in determining the quality of synthetic images. While many studies rely primarily on FID [19, 21, 22, 28, 29], this metric returns a single score that captures only certain aspects of fidelity and diversity. Comprehensive evaluation requires a suite of metrics to assess multiple dimensions of quality, as no single metric suffices [30, 31]. Common metrics such as peak signal-to-noise ratio (PSNR), normalized mean square error (NMSE), and structural similarity index (SSIM) [32], widely used for brain MR image evaluation [33–36], fail to account for the broader distributional relationships between real and synthetic data. A robust evaluation framework should instead assess fidelity (realness of synthetic data), diversity (coverage of real data distribution), and generalization (generation of authentic, non-memorized images). These characteristics are particularly relevant in the context of MRI data, where the complex features demand robust evaluation frameworks [33].

To address these gaps, we investigate the impact of training set size on the performance of StyleGAN2-ADA using a multi-dimensional evaluation framework that assesses the fidelity, diversity, and generalizability of synthetic images. This approach, implemented in an open-source GitHub repository (available at: https://github.com/aiformedresearch/Synthetic_Images_Metrics_Toolkit), is designed for easy adaptability, enabling the community to extend this framework to new datasets and modalities. This aims to provide a nuanced understanding of generative model performance in medical imaging, where data scarcity and privacy constraints are critical concerns.

This study addresses two critical challenges by (1) analyzing the impact of training set size on the performance of StyleGAN2-ADA and (2) emphasizing the need for diverse metrics to comprehensively evaluate synthetic data quality. We trained StyleGAN2-ADA on 2D slices of brain MR images from the OpenBHB dataset [37], which overall contains 3,227 images. To evaluate the impact of training set size, we created three subsets with 1,000, 2,000, and the full set of 3,227 images. Synthetic image quality was assessed both qualitatively and quantitatively, using state-of-the-art metrics for fidelity, diversity, and generalization as implemented in our GitHub repository. The proposed framework may facilitate standardized assessments of generative models in medical imaging research.

## Methods

### Data Source and Preprocessing

This study utilized the OpenBHB dataset [37] (https://baobablab.github.io/bhb/dataset), aggregating T1-weighted brain MRI scans of healthy subjects from 10 publicly available datasets. The dataset, created for a public competition, consists of 3,227 training images, and 757 validation images. We maintained the same split for our experiments, using the 757 images as a test set.

The dataset offers three preprocessing options: CAT12 (optimized for voxel-based morphometry – VBM), FSL (optimized for surface-based morphometry – SBM), and quasi-raw images with minimal preprocessing. For this study, we used quasi-raw images to preserve the inherent characteristics of the MRI scans. The quasi-raw preprocessing pipeline involved bias field correction using ANTS [38], affine registration with FSL FLIRT [39] to the 1*mm*^3^ MNI template (with 9 degrees of freedom, avoiding shearing), and the application of a brain mask to remove non-brain tissues.

To prepare data for our experiments, each volume was normalized to an intensity range of [0,1], and the central axial slice (182 × 218 pixels) was extracted to obtain a 2D image for each subject. The resulting images were zero-padded to the size of 256 × 256 pixels and concatenated into a single NIfTI volume with dimension 256 × 256 × N, where N represents the total number of images.

### Model Training

We employed StyleGAN2-ADA, using the official PyTorch implementation (https://github.com/NVlabs/stylegan2-ada-pytorch). The model was trained with default hyperparameters as proposed by Karras et al. [15], except for removing color-related augmentations (luma flip, hue rotation, and saturation), which are irrelevant for grayscale MR images. The training was conducted for 2,200 kimgs (thousands of images processed in batches), using the full dataset of 3,227 images and subsets of 1,000 and 2,000 images to evaluate the effect of dataset size. Each training session was executed on a single NVIDIA A100 GPU, each configuration requiring approximately 25 days of computation time.

### Quantitative Metrics

**Fig. 1:**
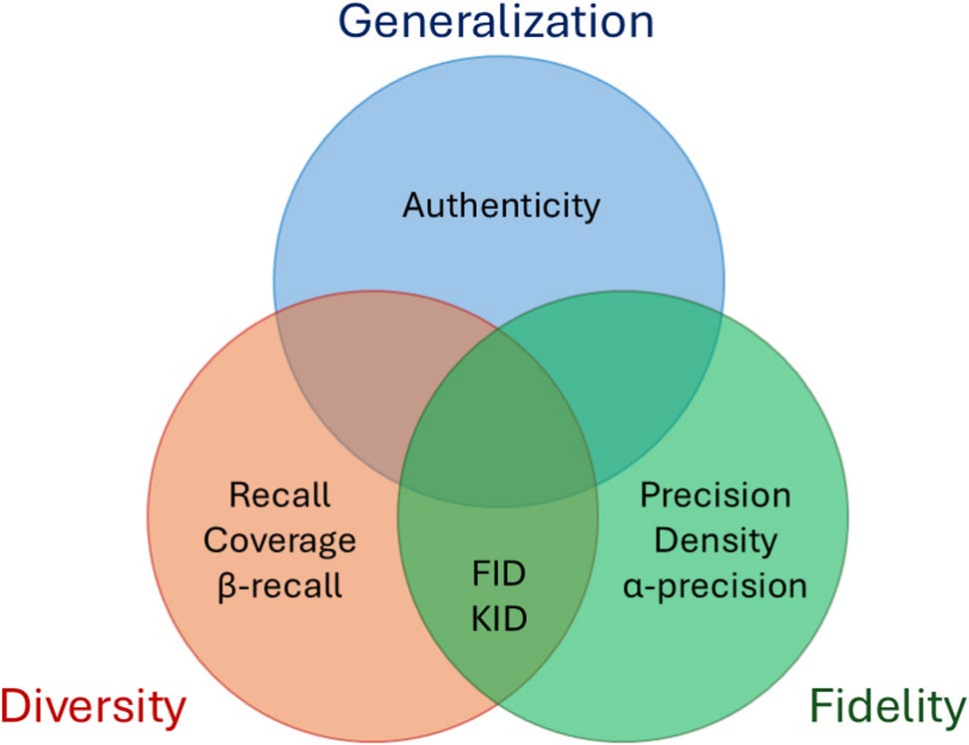
Quantitative metrics grouped by fidelity, diversity, and generalization. This figure was created using PowerPoint

To comprehensively assess synthetic image quality, we computed multiple metrics, summarized in Fig. 1, to assess three aspects: fidelity, diversity, and generalization. Since no single metric captures all facets of generative model performance [30], we integrated implementations from prior studies into a unified GitHub repository. Metrics were computed under two main configurations:

1. *Training set alignment*: synthetic images were compared with the 3,227 images from the training set to evaluate fidelity, diversity, and generalization. To account for the effect of synthetic image quantity, metrics were computed under two sub-configurations:
  - Using 50,000 synthetic images, as suggested by Heusel et al. [40].
  - Using 3,000 synthetic images comparable in size to the real training set.
2. *Unseen data consistency*: synthetic images were compared with 757 unseen real images from the test set to assess robustness and consistency with out-of-sample data. Similar to the first configuration, metrics were computed under two sub-configurations:
  - Using 50,000 synthetic images, as recommended by Heusel et al. [40].
  - Using 700 synthetic images, matching the size of the real test set.

#### Fréchet Inception Distance (FID)

FID [40] computes the Fréchet (Wasserstein-2) distance between the distributions (estimated as Gaussian) of real and synthetic images in the feature space of an Inception network. A lower FID score reflects closer alignment between real and synthetic distributions; therefore, it is influenced by fidelity and diversity. FID is considered the standard metric for comparing the quality of synthetic images generated by generative models [30] and has shown consistency with human perceptual evaluation of medical images [19]. However, FID is biased by the number of images involved in the computation [41].

#### Kernel Inception Distance (KID)

KID [41] provides an unbiased alternative to FID by using a polynomial kernel to estimate the similarity between real and synthetic image distributions in the feature space of an Inception Net. Like FID, it captures fidelity and diversity, with a lower score indicating better alignment.

#### Precision and Recall

Precision and recall quantify fidelity and diversity separately using the feature space of a pre-trained VGG-16 classifier [42]. Real and synthetic image distributions are modeled as manifolds (by considering the k^th^ nearest feature vector of each embedded image), and the metrics measure the overlap between these distributions. Precision is computed by counting the fraction of generated samples covered by the real data manifold, assessing the fidelity (i.e., the realness) of the synthetic images. Similarly, recall counts the fraction of the real data manifold covered by the generator, assessing the diversity of the generative model. Both scores range from 0 to 1, with 1 indicating the ideal performance.

#### Density and Coverage

Density and coverage have been proposed by Naeem et al. to improve precision and recall, which are vulnerable to outliers due to the way the manifold is obtained [43]. To overcome this, density estimates the fidelity by counting how many real-sample neighborhood spheres contain synthetic samples. On the other hand, coverage assesses the diversity by counting the fraction of real samples whose neighborhood spheres contain at least one synthetic sample, building the nearest-neighbor manifold around real data. These scores range in [0,1], with 1 being the ideal value.

#### α-precision, β-recall, and Authenticity

α-precision and β-recall enhance traditional precision and recall by addressing their sensitivity to outliers and dependency on the domain of the pre-trained model [44]. To compute these metrics, images are initially embedded through an Inception V3 network pre-trained on ImageNet. The resulting embeddings are then mapped into a hypersphere using a one-class (OC) classifier. The OC classifier is trained to concentrate most of the samples around the center of the hypersphere, where “typical” samples are located, while positioning outliers closer to the boundaries. In this transformed space, precision and recall are computed iteratively by varying the thresholds that define “typical” samples, controlled by the parameters α and β. The iterative process generates curves representing the variation in precision and recall as the definition of"typical"changes. The final scores, which range in [0,1], are obtained considering the area under those curves, with higher values indicating better performance in terms of fidelity (α-precision) and diversity (β-recall).

Authenticity measures the fraction of synthetic data not memorized from the training set [44]. To compute this metric, real and synthetic images are first embedded using the OC classifier. For each real image, the metric checks if its nearest neighbor is another real or synthetic image. If a synthetic image is closer to a real image than any other real image, that real image is considered memorized by the model. The authenticity score ranges from 0 to 1, with higher scores indicating better generalization. To calculate this, we compared all real images against batches of synthetic images of the same size, repeating the process until we covered 50,000 synthetic images. The average score across all batches was used as the final authenticity value.

### Qualitative Evaluation

To qualitatively evaluate the generative model, we examined fidelity, diversity, and generalization, mirroring the aspects assessed quantitatively. **Fidelity** was assessed using a visual Turing test, where two expert observers (#1, M.M. and #2, C.T., with 35 and 30 years of experience, respectively) classified 100 images (50 real, 50 synthetic) presented in random order as real or fake. Crucially, the observers were blinded to the fact that the dataset contained an equal proportion of real and synthetic images, ensuring an unbiased evaluation. The experiment was repeated for each training set size using a custom-built tool that enabled adjustment of window/level settings [45].

For each classification, we recorded the number of real images correctly classified as real (true positive – TP), real images misclassified as synthetic (false negative – FN), synthetic images correctly classified as synthetic (true negative – TN), and synthetic images misclassified as real (false positive – FP). From these values, we calculated the following metrics:

$$Accuracy= \frac{TP+TN}{TP+TN+FP+FN}$$

$$Sensitivity=\frac{TP}{TP+FN}$$

$$Specificity= \frac{TN}{TN+FP}$$

**Diversity** was assessed using t-SNE visualization, which projected real and synthetic images into two dimensions. These visualizations were generated using the Compyda web service [46] (https://cbia.fi.muni.cz/compyda/), using all the 3,227 real images from the training set and 5,000 synthetic images generated by StyleGAN2-ADA when trained on the entire dataset. The analysis was repeated using the 757 real images from the test set to evaluate consistency across unseen data.

**Generalization** was assessed using k-NN analysis. Real and synthetic images were embedded using a pre-trained VGG-16 network. Cosine similarity was then calculated between the embedding of each real image and 50,000 synthetic images. For each real image, the k^th^ (k = 4) closest synthetic images were identified and ranked by similarity. To visualize the results, the two real images with the highest similarity to any synthetic image were displayed alongside their four closest synthetic counterparts.

## Results

**Fig. 2:**
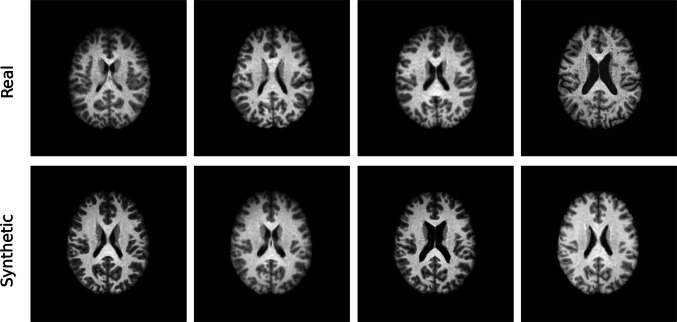
Comparison of synthetic and real images: the bottom row displays real images randomly sampled from the training set, while the top row showcases synthetic images generated by StyleGAN2-ADA when trained on the entire dataset (3,227 images). This figure was created using Python

**Fig. 3:**
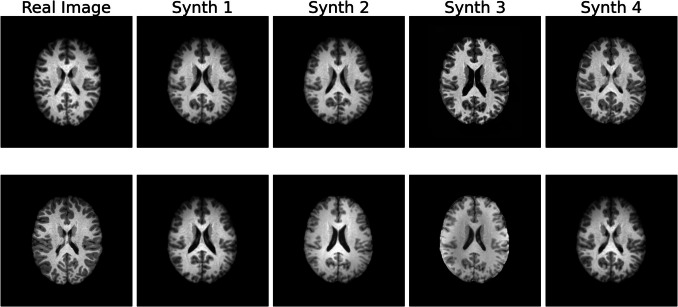
k-NN analysis. Visualization of the 4-nearest neighbors (k-NN) for real images from the training set. The first column displays the two real images that exhibit the highest cosine similarity with any synthetic sample. Each subsequent column shows the top four synthetic images (out of 50,000 generated samples) ranked by their similarity to the corresponding real image. This layout highlights the ability of the model to generate synthetic images that closely resemble real data without memorizing samples from the training set. This figure was created using Python

**Fig. 4:**
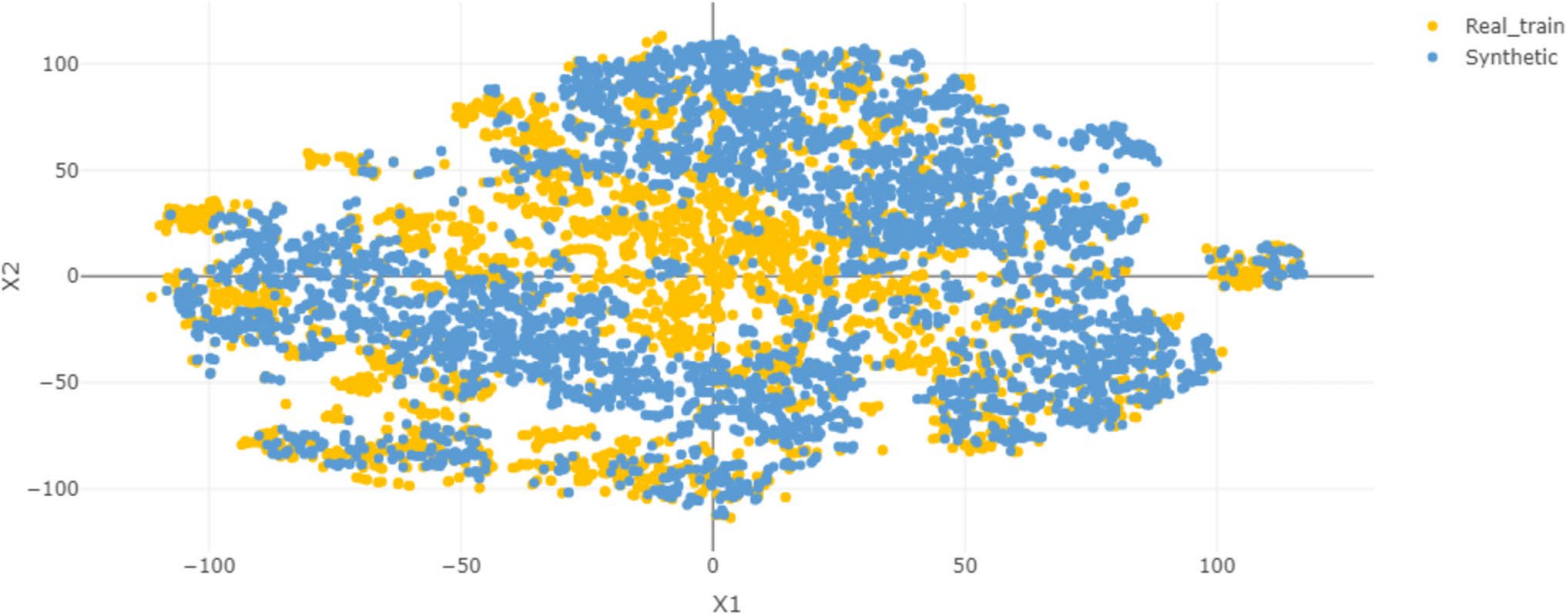
T-SNE visualization of 5,000 synthetic images (blue) compared with all the real images from the training set (orange). This figure was created using the Compyda web service

The results of the qualitative evaluation conducted through the visual Turing test are collected in Table 1. Figure 2 provides examples of randomly sampled real and synthetic images generated by StyleGAN2-ADA trained on the full dataset. The model demonstrated strong generalization, producing synthetic images not memorized from the training set, as revealed from the k-NN analysis shown in Fig. 3. However, the t-SNE visualization (Fig. 4) of 5,000 synthetic images compared with the training set indicates that the model is unable to capture the entire real data distribution. All qualitative evaluations were conducted on images generated by the model trained on the full dataset to streamline the analysis.

**Table 1:** Results of the visual Turing test. The classification was performed by two experts for each training set size (TP, true positives; FN, false negatives; TN, true negatives; FP, false positives)

Observer	Training set size	TP	FP	TN	FN	Accuracy	Sensitivity	Specificity
#1	1,000	36	33	17	14	0.53	0.72	0.34
	2,000	36	31	19	14	0.55	0.53	0.38
	3,227	32	31	19	18	0.51	0.64	0.38
#2	1,000	18	24	26	32	0.44	0.32	0.52
	2,000	26	33	17	24	0.43	0.52	0.34
	3,227	29	31	19	21	0.48	0.58	0.38

Quantitative metrics were computed for each training set size by comparing synthetic images against both the training set (to evaluate memorization) and an unseen test set (to assess generalization). Unless otherwise specified, results discussed in this paper refer to metrics computed between the training set and 50,000 synthetic images.

**Fig. 5:**
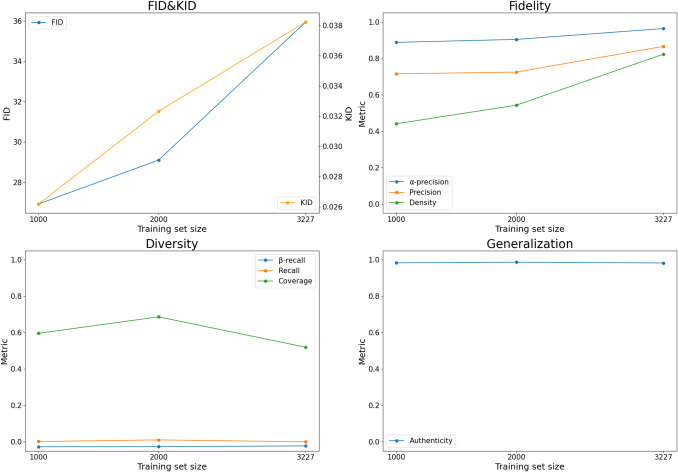
Quantitative metrics for increasing training set size. The y-axis, labeled “Metric,” represents the values of the specific metrics identified in the legend. This figure was created using Python

The quantitative results aligned with the qualitative findings, confirming that the images generated by StyleGAN2-ADA are realistic, not memorized from training data, but exhibit limited diversity. The metric scores computed between the full training set (3,227 images) and 50,000 synthetic images are collected in Supplementary Table 1 and visualized in Fig. 5, grouped by category (fidelity, diversity, and generalization). Notably, the metrics highlighted a positive correlation between training set size and fidelity, demonstrating a slightly improved realism with larger training datasets. Some of the diversity metrics, such as coverage, were notably sensitive to the number of images used in the computation. This effect is evident when comparing the same real training images with a reduced set of 3,000 synthetic images (Supplementary Table 2, Supplementary Fig. 1).

To further assess performance on unseen data, metrics were calculated using 757 real test set images and either 50,000 or 700 synthetic images. The results, detailed in Supplementary Tables 3 and 4 and visualized in Supplementary Fig. 2 and 3, reinforce the finding that the model generates realistic yet insufficiently diverse images across dataset configurations. They also highlight the sensitivity of certain diversity metrics, such as β-recall and coverage, to the number of images used in the evaluation.

## Discussion

This study investigates the impact of training set size on the quality of synthetic images generated by StyleGAN2-ADA, trained on the OpenBHB dataset to generate 2D slices of brain MRI from healthy subjects. Three training set sizes (1,000, 2,000, and 3,227 images) were analyzed using both qualitative and quantitative methods to assess fidelity, diversity, and generalization.

### Effect of Training Set Size

Consistent with findings by Zoghby et al. [27], training set size had minimal influence on the overall quality of the synthetic images. However, it is important to note that their work focused on image-to-image translation (transforming one image type into another), whereas our study investigates unconditional image synthesis, where entirely new images are generated from a learned distribution. Despite this methodological difference, our results reinforce their central observation regarding the limited impact of dataset size while extending its relevance to the domain of generative image synthesis.

In our study, while fidelity metrics, as shown in Fig. 5, indicated a slight improvement in image realism with larger training set sizes, this trend was not observed in the visual Turing test results conducted by expert radiologists, whose classification accuracy remained near chance level regardless of the training set size. In contrast, diversity metrics remained consistently low across all configurations, pointing to persistent mode collapse regardless of the training set size. Generalization metrics remained optimal even with the smallest datasets, suggesting that StyleGAN2-ADA effectively avoids overfitting even when trained on limited data.

Our findings highlight that the adaptive discriminator augmentations in StyleGAN2-ADA enable the generation of highly realistic, non-memorized images, even with small datasets, with a slight increase in realism as the training set size increases. However, the mechanisms implemented in StyleGANs to address mode collapse appear insufficient in low-data regimes. This suggests that additional strategies may be necessary to enhance variation in generated images when training data is limited. Our analysis underscores the need for further research into architectural modifications or training techniques that can better maintain diversity in generated outputs, particularly in medical imaging where data scarcity is a common challenge.

The apparent inconsistency between FID/KID scores and fidelity metrics is likely influenced by the low diversity of the generated images, as discussed further below. These results emphasize the need for comprehensive evaluation protocols incorporating domain-specific fidelity and diversity metrics to complement composite metrics like FID and KID.

### Synthetic Data Quality

#### FID and KID

FID and KID, widely used composite metrics for assessing generative model quality, ranged from 26.92 to 35.94 and 0.026 to 0.038, respectively. Analysis revealed that both FID and KID scores increased modestly when fewer synthetic images were used in the computation (as observed when comparing results from Supplementary Tables 1 to 2, and 3 to 4). Notably, a significant increase in their values occurred when the number of real images in the computation was reduced (transitioning from Supplementary Tables 1 and 2 to Supplementary Tables 3 and 4). This sensitivity to dataset configuration emphasizes the limitations of these composite metrics in reliably capturing image quality across different evaluation settings. Hence, these findings highlight the importance of supplementing FID and KID with domain-specific metrics to achieve a more comprehensive and accurate assessment of fidelity and diversity.

#### Fidelity

Visual inspection (Fig. 2) indicates that synthetic images produced by StyleGAN2-ADA are realistic, albeit some blurring artifacts are shared with the training data. The visual Turing test revealed that even expert observers had difficulty in distinguishing between real and synthetic images, with classification accuracies close to 50%. Interestingly, the size of the training set had no substantial impact on the accuracy, sensitivity, or specificity of either observer.

Quantitatively, fidelity was assessed using precision, density, and α-precision, providing complementary insights into the quality of synthetic data. Precision increased from 71.5% to 86.5% with larger training sets, reflecting an improved ability of synthetic images to reside within the real data manifold. Density, which measures the portion of the real data manifold covered by the synthetic images, increases from 44% to 82.3% when increasing the training set size. The gap between precision and density suggests that outliers in the real data may have inflated the manifold size, inflating the value of precision. α-Precision, also designed to address outlier sensitivity, exhibited robust values (88.7% to 90.4%) across all training set sizes, further validating these findings.

Fidelity metrics remained consistent regardless of the number of synthetic images used in the computation (see Supplementary Tables 1 and 2) and yielded comparable values when evaluated against the test set (Supplementary Tables 3 and 4). This consistency demonstrates the robustness of these metrics and highlights the ability of StyleGAN2-ADA to effectively generate realistic data closely aligned with the real data distribution.

#### Diversity

Diversity was assessed qualitatively using t-SNE visualization (Fig. 4) and quantitatively with recall, coverage, and β-recall metrics. The t-SNE plots revealed gaps in the embedding space of synthetic data compared with real data, suggesting mode collapse – a failure to capture all modes of real data distribution.

Recall and β-recall scored near zero, indicating that only a small fraction of real data points falls within the synthetic data manifold. Coverage, representing the proportion of the real data’s neighborhood spheres containing synthetic samples, ranged from 51.9% to 68.6% when computed with 50,000 synthetic images. However, coverage dropped to values in 30.9–38.9% when only 3,000 synthetic images were used for the evaluation – see Supplementary Table 2 and Supplementary Fig. 1. This drop highlights the sensitivity of coverage to the number of synthetic samples, likely inflating results when synthetic samples vastly outnumber real ones.

Interestingly, when compared to the test set, diversity metrics such as coverage and β-recall improved (Supplementary Table 3), suggesting that StyleGAN2-ADA captures broader patterns beyond the training set. However, diversity metrics dropped when the number of synthetic samples was reduced to match the size of the test set (Supplementary Table 4), reaffirming their dependency on synthetic set size. These findings confirm that StyleGAN2-ADA suffers from mode collapse, and diversity metrics such as coverage and β-recall should be interpreted with caution, particularly when synthetic samples vastly outnumber real ones.

#### Generalization

Generalization was evaluated through k-NN analysis and authenticity metrics, which collectively indicated that StyleGAN2-ADA does not memorize training data. In the k-NN analysis (Fig. 3), synthetic images most similar to real images were visually distinct from their nearest real neighbors, demonstrating that the model generates new data rather than replicating training samples.

Quantitatively, authenticity analysis showed that 98% of synthetic images were farther from the nearest real image than real images were from each other, supporting the conclusion that the model generates new data. Notably, these results held across all training set sizes, affirming StyleGAN2-ADA’s robustness as a privacy-preserving generative model.

### Broader Implications and Limitations

This study highlights the need for standardized evaluation protocols for generative models, particularly in medical imaging. To this end, we compiled state-of-the-art metrics into a publicly available GitHub repository to promote reproducibility and consistency in evaluation practices.

While StyleGAN2-ADA demonstrated strong fidelity and generalization, its diversity limitations underscore the persistent challenge of mode collapse in GANs. Future research should explore alternative generative architectures, such as diffusion models, which may address this limitation. Additionally, this study focused on 2D slices of brain MRI, while clinical applications often require 3D data. Extending the analysis to volumetric synthesis represents a crucial step toward clinical applicability, though it is important to note that such an extension would result in a significant increase in computational demands.

A limitation of our work is that we did not explore the causal relationships between network architecture, dataset size, and quality metrics. Future research could investigate how specific network components interact with training data volume to influence different quality dimensions through controlled ablation studies.

Lastly, while this work primarily evaluated image quality, future evaluations should incorporate the *utility* of synthetic data for downstream tasks, such as classification or regression [47], to fully understand its practical value.

## Conclusion

This study demonstrates that StyleGAN2-ADA generates realistic and novel brain MR images, capable of deceiving even expert radiologists, with high fidelity and strong generalization, regardless of training set size. However, the diversity of the model exhibits significant limitations, failing to fully capture the variability of real data due to mode collapse. Notably, increasing the training set size alone does not mitigate mode collapse, suggesting the need for alternative strategies.

The results also reveal the sensitivity of diversity metrics, such as coverage and β-recall, to synthetic set size. When synthetic samples vastly outnumber real ones, these metrics may overestimate diversity. This underscores the importance of interpreting such metrics alongside complementary indicators to ensure robust evaluations.

Future research should explore alternative generative models, investigate the causal relationships between network architecture and performance across different dataset sizes, and include downstream task evaluations to comprehensively assess the utility of synthetic data in clinical and research contexts. By addressing these challenges, generative models can better meet the requirements of medical imaging applications, ultimately enhancing their reliability and clinical relevance.

## Supplementary Information

Below is the link to the electronic supplementary material.

Supplementary file1 (DOCX 920 KB)

## Data Availability

The dataset used for the training of the model can be accessed at https://ieee-dataport.org/open-access/openbhb-multi-site-brain-mri-dataset-age-prediction-and-debiasing.

## Code Availability

The source code used to compute the quantitative metrics has been made available at the public GitHub repository: https://github.com/aiformedresearch/Synthetic_Images_Metrics_Toolkit. Additionally, a Docker container is available at https://hub.docker.com/r/aiformedresearch/metrics_toolkit.
