# Supplementary material for: Generating Brain MRI with StyleGAN2-ADA: The Effect of the Training Set Size on the Quality of Synthetic Images
Source: J Imaging Inform Med. 2025 Sep 23;39(3):2319–29. doi: 10.1007/s10278-025-01536-0 (PMC13230348; doi:10.1007/s10278-025-01536-0)

Supplementary material to *Generating brain MRI with StyleGAN2-ADA: the effect of the training set size on the quality of synthetic images*

**Supplementary Tables**

**Supplementary Table 1**

Metrics were computed between all the 3,227 real *training* set images and 50,000 synthetic images. Results are shown for different training set sizes (1,000, 2,000, and 3,227 images). Metrics are grouped into fidelity, diversity, and generalization categories. Arrows (↑/↓) indicate whether higher or lower values are optimal for each metric.

| Training set size | | |  |  |  |  |
| --- | --- | --- | --- | --- | --- | --- |
| Metric | 1000 | 2000 | 3227 | Range | |  |
|  | FID | 26.926 | 29.099 | 35.945 | [0, ∞) | ↓ |
|  | KID | 0.0261 | 0.0323 | 0.0382 |  |  |
| Fidelity | Precision | 0.7152 | 0.7246 | 0.8658 | [0,1] | ↑ |
|  | Density | 0.4407 | 0.5428 | 0.8230 |  |  |
|  | α-precision | 0.8879 | 0.9041 | 0.8900 |  |  |
| Diversity | Recall | 0.0015 | 0.0105 | 0.0000 |  |  |
|  | Coverage | 0.5962 | 0.6867 | 0.5196 |  |  |
|  | β-recall | 0.0000 | 0.0000 | 0.0000 |  |  |
| Generalization | Authenticity | 0.9832 | 0.9867 | 0.9893 |  |  |

**Supplementary Table 2**

Metrics were computed between all the 3,227 real *training* set images and 3,000 synthetic images. Results are shown for different training set sizes (1,000, 2,000, and 3,227 images). Metrics are grouped into fidelity, diversity, and generalization categories. Arrows (↑/↓) indicate whether higher or lower values are optimal for each metric.

| Training set size | | |  |  |  |  |
| --- | --- | --- | --- | --- | --- | --- |
| Metric | 1000 | 2000 | 3227 | Range | |  |
|  | FID | 28.094 | 29.684 | 36.483 | [0, ∞) | ↓ |
|  | KID | 0.0254 | 0.0318 | 0.0386 |  |  |
| Fidelity | Precision | 0.7096 | 0.7260 | 0.8710 | [0,1] | ↑ |
|  | Density | 0.4438 | 0.5491 | 0.8175 |  |  |
|  | α-precision | 0.8983 | 0.9162 | 0.9645 |  |  |
| Diversity | Recall | 0.0251 | 0.0529 | 0.0000 |  |  |
|  | Coverage | 0.3126 | 0.3895 | 0.3098 |  |  |
|  | β-recall | 0.0000 | 0.0000 | 0.0000 |  |  |
| Generalization | Authenticity | 0.9865 | 0.9867 | 0.9918 |  |  |

**Supplementary Table 3**

Metrics were computed between all the 757 real *test* set images and 50,000 synthetic images. Results are shown for different training set sizes (1,000, 2,000, and 3,227 images). Metrics are grouped into fidelity and diversity categories. Arrows (↑/↓) indicate whether higher or lower values are optimal for each metric.

| Training set size | | |  |  |  |  |
| --- | --- | --- | --- | --- | --- | --- |
| Metric | 1000 | 2000 | 3227 | Range | |  |
|  | FID | 148.82 | 141.49 | 166.64 | [0, ∞) | ↓ |
|  | KID | 0.2098 | 0.1965 | 0.2489 |  |  |
| Fidelity | Precision | 0.7679 | 0.7736 | 0.8884 | [0,1] | ↑ |
|  | Density | 0.4815 | 0.5658 | 0.7412 |  |  |
|  | α-precision | 0.8318 | 0.8727 | 0.8525 |  |  |
| Diversity | Recall | 0.0013 | 0.0158 | 0.0000 |  |  |
|  | Coverage | 0.7886 | 0.8573 | 0.7014 |  |  |
|  | β-recall | 0.7108 | 0.7042 | 0.5778 |  |  |

**Supplementary Table 4**

Metrics were computed between all the 757 real *test* set images and 700 synthetic images. Results are shown for different training set sizes (1,000, 2,000, and 3,227 images). Metrics are grouped into fidelity and diversity categories. Arrows (↑/↓) indicate whether higher or lower values are optimal for each metric.

| Training set size | | |  |  |  |  |
| --- | --- | --- | --- | --- | --- | --- |
| Metric | 1000 | 2000 | 3227 | Range | |  |
|  | FID | 150.65 | 143.22 | 169.11 | [0, ∞) | ↓ |
|  | KID | 0.2103 | 0.1930 | 0.2474 |  |  |
| Fidelity | Precision | 0.7557 | 0.7871 | 0.8814 | [0,1] | ↑ |
|  | Density | 0.4768 | 0.5602 | 0.7194 |  |  |
|  | α-precision | 0.8336 | 0.8728 | 0.8253 |  |  |
| Diversity | Recall | 0.0541 | 0.1624 | 0.0105 |  |  |
|  | Coverage | 0.3632 | 0.4161 | 0.3447 |  |  |
|  | β-recall | 0.3415 | 0.3493 | 0.2918 |  |  |

**Supplementary Figures**

**Supplementary Fig. 1** Metrics computed between 3,000 synthetic images and all the 3,227 images from the training set. This figure was created using Python


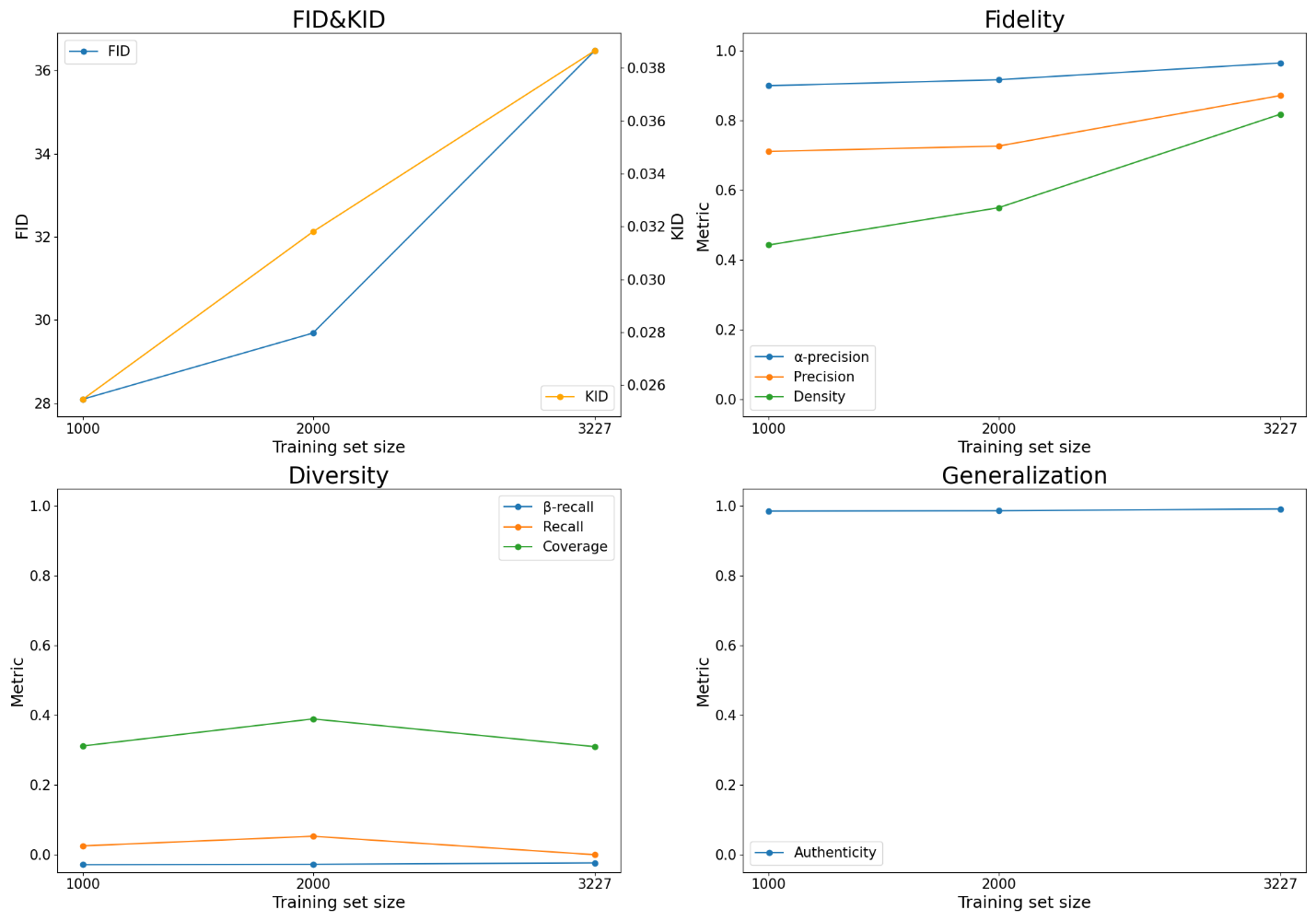


**Supplementary Fig. 2** Metrics computed between 50,000 synthetic images and 757 real images from the test set. This figure was created using Python


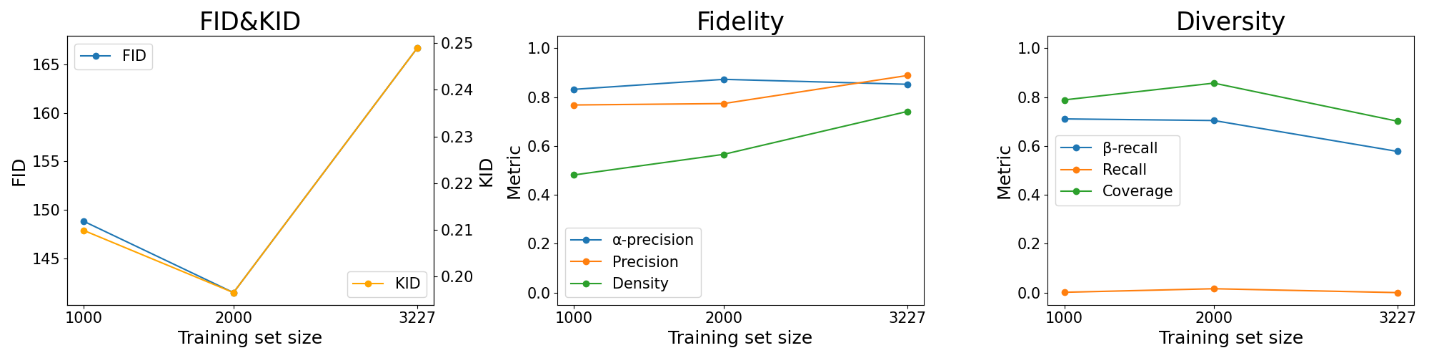


**Supplementary Fig. 3** Metrics computed between 700 synthetic images and 757 real images from the test set. This figure was created using Python


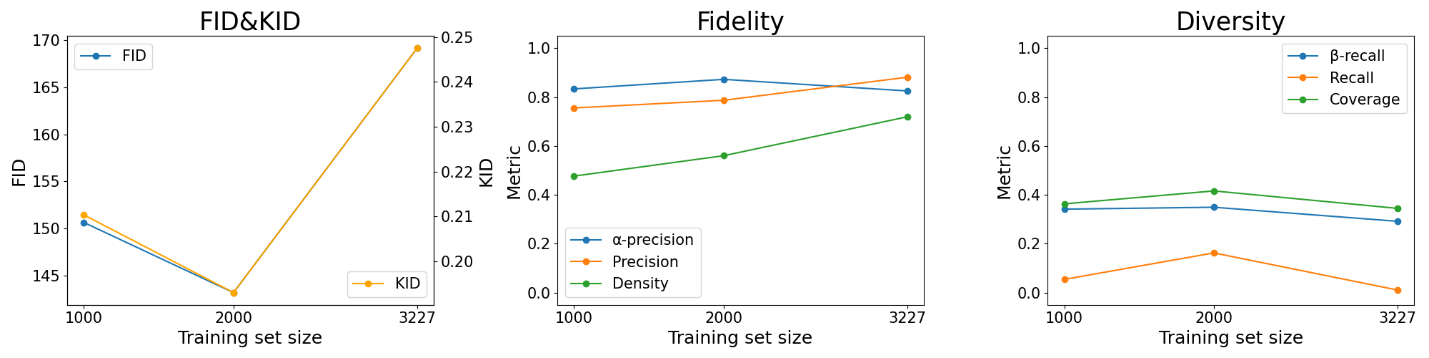

Supplement: Supplementary file 1 — Supplementary file1 (DOCX 920 KB) [file 10278_2025_1536_MOESM1_ESM.docx]
